# The Artificial Promoter *rMdAG2I* Confers Flower-specific Activity in *Malus*

**DOI:** 10.3390/ijms20184551

**Published:** 2019-09-13

**Authors:** Haiqiang Dong, Lanxin Liu, Xingqiang Fan, Sumeera Asghar, Yi Li, Yi Wang, Xuefeng Xu, Ting Wu, Xinzhong Zhang, Changpeng Qiu, Zhenhai Han, Wei Li

**Affiliations:** 1 College of Horticulture, China Agricultural University, Beijing 100193, China; dhq11743@126.com (H.D.); liulanxin8786@163.com (L.L.); fanxingqiang@126.com (X.F.); sumeraroy211@gmail.com (S.A.); wangyi@cau.edu.cn (Y.W.); xuefengx@cau.edu.cn (X.X.); wuting@cau.edu.cn (T.W.); zhangxinzhong999@126.com (X.Z.); changpengqiu@163.com (C.Q.); 2 Department of Plant Science and Landscape Architecture, University of Connecticut, Storrs, CT 06269, USA; yi.li@uconn.edu.cn

**Keywords:** flower-specific promoter, *Malus*, *AGAMOUS*, intron

## Abstract

Genetic modifications of floral organs are important in the breeding of *Malus* species. Flower-specific promoters can be used to improve floral organs specifically, without affecting vegetative organs, and therefore developing such promoters is highly desirable. Here, we characterized two paralogs of the *Arabidopsis thaliana* gene *AGAMOUS (AG)* from *Malus domestica* (apple): *MdAG1* and *MdAG2*. We then isolated the second-intron sequences for both genes, and created four artificial promoters by fusing each intron sequence to a minimal *35S* promoter sequence in both the forward and reverse directions. When transferred into tobacco (*Nicotiana benthamiana*) by *Agrobacterium tumefaciens*-mediated stable transformation, one promoter, *rMdAG2I*, exhibited activity specifically in flowers, whereas the other three also showed detectable activity in vegetative organs. A test of the four promoters’ activities in the ornamental species *Malus micromalus* by *Agrobacterium*-mediated transient transformation showed that, as in tobacco, only *rMdAG2I* exhibited a flower-specific expression pattern. Through particle bombardment transformation, we demonstrated that *rMdAG2I* also had flower-specific activity in the apple cultivar ‘Golden Delicious’. The flower-specific promoter *rMdAG2I*, derived from *M. domestica*, thus has great potential for use in improving the floral characteristics of ornamental plants, especially the *Malus* species.

## 1. Introduction

The genus *Malus* comprises more than 30 species with both ornamental and edible uses [1]. Flowering crabapples belonging to this genus are widely used in landscape gardening, due to their varied and attractive flower color, size and fragrance [2]. *Malus* plants are always self-sterile, requiring cross-pollination by insects, such as bees, which is important for fruit production in domesticated apples [3,4,5,6,7]. Flowers are key reproductive organs, because they play important roles in attracting pollinating insects. Therefore, *Malus* flowers are important both for the plants’ value as ornamentals and for pollination purposes [8,9,10,11]. Improvement of flower traits is a crucial target in breeding *Malus* species.

Transgenic technology has been used in the genetic improvement of a series of flower traits, including flower color, size and stress resistance [12,13,14]. However, constitutive expression of target genes may have undesirable effects on the vegetative organs. For instance, although cytokinins can be used to regulate flower size, constitutive regulation of cytokinins would also affect vegetative growth [15,16]. A flower-specific promoter could be of great utility by enabling the application of transgenic methods to improve flower traits, specifically without affecting vegetative growth.

In *Arabidopsis thaliana*, the gene *AGAMOUS* (*AG*), initially expressed in the third stage of flower development, is the key factor determining the identity of carpel and stamen [17,18]. The second intron of *AGAMOUS* contains *cis*-regulatory elements influencing the flower-specific expression of target genes [19,20]. In woody plants, Li et al. cloned the second intron of one of the poplar *AG* paralogs, *PTAG2*, and demonstrated that, when fused with the minimal *35S* promoter, the reverse version of the *PTAG2* second intron exhibits flower-specific activity [21]. However, a flower-specific promoter derived from the *Arabidopsis AG* second intron does not retain its specificity in tobacco plants [21]. Therefore, isolating the second intron from *Malus* paralogs of *AG* as a tool for the transgenic improvement of flowers in the *Malus* species is needed.

In *M. domestica*, two *MADS*-box genes, *MdMADS15* and *MdMADS22*, have been reported as paralogs of *AGAMOUS* [22]. Furthermore, RNA interference (RNAi) experiments indicate that RNA interference against two apple *AG* paralogs cause male and female sterility, suggesting these genes play important roles in both male and female floral structures [23], similar to the *AG* genes in *Arabidopsis* [24,25,26]. In this study, we cloned the second introns of two *AG* paralogs, and characterized the promoter activity by fusing them to a β-glucuronidase gene (*uidA, or GUS*) in both forward and reverse directions. We reported that the reverse version of the second intron of *MdMADS22* drove the flower-specific expression of target genes both in tobacco and in two *Malus* species, *Malus domestica* (apple) and the ornamental *Malus micromalus* (midget crabapple). This artificial promoter could thus serve as a tool for the genetic manipulation of *Malus* species and other flowering plants.

## 2. Results and Discussion

### 2.1. Isolation and Expression Analysis of MdAG1 and MdAG2

**Figure 1:**
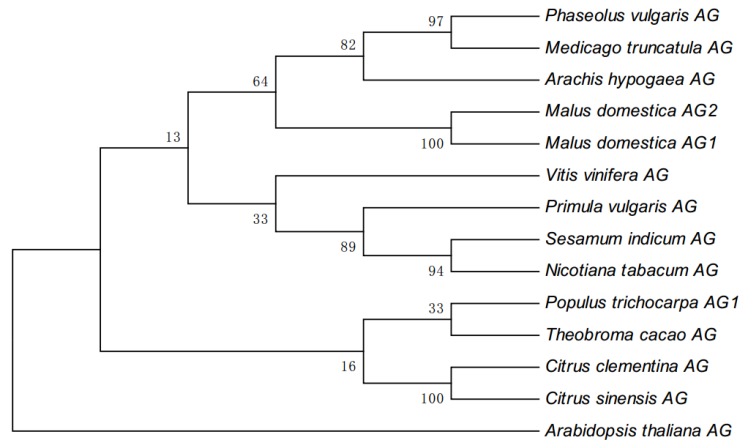
Phylogenetic analysis of *AG* protein sequences. The phylogenetic tree was constructed using the neighbor-joining method of MEGA 6.0 software based on the protein sequences of *Phaseolus vulgaris AG* (Phvul.006G169600), *Medicago truncatula AG* (XP_013462626.1), *Arachis hypogaea AG* (XP_025640343.1), *Malus domestica AG2* (MDP0000250080), *Malus domestica AG1* (MDP0000324166), *Vitis vinifera AG* (NP_001268097.1), *Primula vulgaris AG* (AZZ09252.1), *Sesamum indicum AG* (AIS82595.1), *Nicotiana tabacum AG* (NP_001312829.1), *Populus trichocarpa AG1* (AAC06237.1), *Theobroma cacao AG* (XP_007025252.1), *Citrus clementina AG* (XP_006449577.1), *Citrus sinensis AG* (XP_024950555.1) and *Arabidopsis thaliana AG* (NP_001328877.1). Values at nodes indicate bootstrap percentages.

To confirm the apple *AGAMOUS* (*AG*) paralogs, we performed a BLAST analysis of the protein sequence of *Arabidopsis AG* against the protein database of the *M. domestica* variety ‘Golden Delicious’. We identified two *AG* paralogs, which we named *MdAG1* and *MdAG2*. *MdAG1* and *MdAG2* were reported previously as *MdMADS15* and *MdMADS22*, respectively [22,23]. A phylogenetic tree constructed based on the protein sequences of various well-studied *AG* genes revealed that *MdAG1* and *MdAG2* were clustered closely, compared to other *AG* genes (Figure 1).

**Figure 2:**
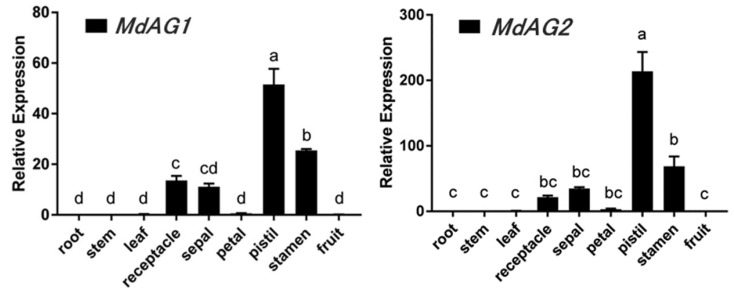
Relative expression levels of *MdAG1* and *MdAG2* in various tissues of *Malus domestica* ‘Golden Delicious’. Expression levels of *MdActin* were used as an internal reference. Data represent means of three biological replicates ± SE. Bars with different letters are significant different at *p* < 0.05 based on one-way analysis of variance (ANOVA).

Next, we characterized the expression patterns of *MdAG1* and *MdAG2* in *M. domestica*. Both *MdAG1* and *MdAG2* exhibited similar floral-organ-predominant expression patterns (Figure 2). Both genes had relatively high expression in pistils and stamens, whereas in vegetative organs such as root, stem and leaf, their expression levels were rarely detectable. In many *Malus* species, fruits develop mainly from receptacles [27]. Interestingly, although both *MdAG1* and *MdAG2* were expressed in receptacles, we did not detect their expression in fruits, indicating that these genes may regulate fruit development in a dynamic fashion [28].

### 2.2. Cloning and Analysis of the Second Introns of MdAG1 and MdAG2

**Figure 3:**
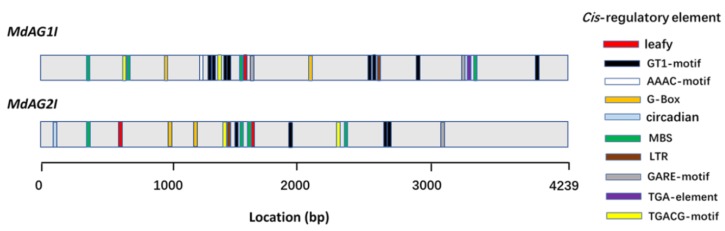
Distributions of ten types of predicted cis-regulatory elements in the second introns of *MdAG1* and *MdAG2*. The different distribution modes may cause different activities when these are used as promoters.

We cloned the sequences of the second introns of *MdAG1* and *MdAG2* and named them as *MdAG1I* (MN403053) and *MdAG2I* (MN401764), respectively. The two sequences showed 87% identity and were 4,239 bp and 4,236 bp in length, respectively. We further analyzed the putative *cis*-regulatory elements of both intron sequences using PlantCARE [29]. The distributions of some known *cis*-regulatory elements in both intron sequences are shown in Figure 3. Both *MdAG1I* and *MdAG2I* have core *cis*-regulatory elements, such as TATA-box and CAAT-box elements. For instance, *MdAG1I* has 25 TATA boxes and 24 CAAT boxes, while *MdAG2I* has 30 TATA boxes and 28 CAAT boxes. In addition, we identified a series of hormone- and environmental-signal-related *cis*-regulatory elements, including three light-responsive elements (GT1 motif, AAAC motif, and G box), one circadian control element (circadian), one drought-responsive element (MBS), one low-temperature-responsive element (LTR), three hormone-related *cis*-regulatory elements (GARE motif, TGA element, and TGACG motif), and one leafy (LFY)-binding site. The LFY-binding site is reported to bind with *AG* second-intron sequences, a process that plays important roles in maintaining *AG* expression in floral organs [30,31,32]. We identified one LFY-binding site in *MdAG1I* and two in *MdAG2I*, and they were also distributed differently within the two intron sequences. These differences may result in different expression patterns when the introns act as promoters.

### 2.3. Floral-Specific Expression of rMdAG2I::GUS in Tobacco

**Figure 4:**
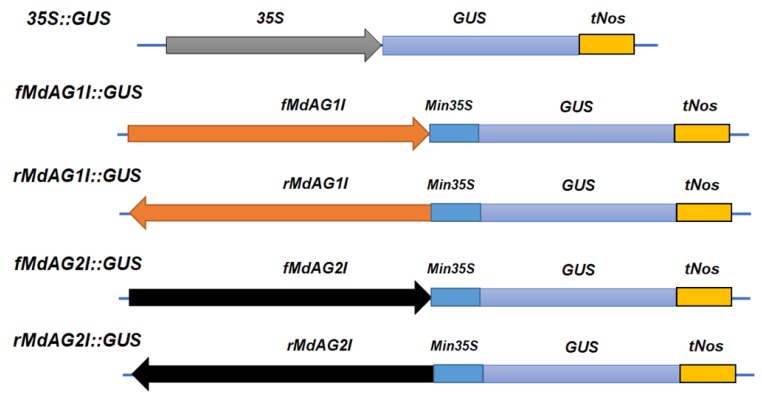
Gene constructs used for tobacco and *Malus* transformation. *35S*, cauliflower mosaic virus 35S gene promoter sequence; *GUS*, coding sequence of the β-glucuronidase gene; *tNos*, nopaline synthase terminator; *fMdAG1I*, forward orientation of the second intron of *Malus AGAMOUS1* (*MdAG1*); *rMdAG1I*, reverse orientation of the second intron of *MdAG1*; *fMdAG2I*, forward orientation of the second intron of *MdAG2*; *rMdAG2I*, reverse orientation of the second intron of *MdAG2*; *Min35S*, 60 bases of the *35S* gene promoter sequence.

To characterize the promoter activity of the two intron sequences, we fused each sequence with the β-glucuronidase (*GUS*) gene. It has been reported that the directions of intron sequences may direct different expression patterns [21]. Therefore, we created four different constructs by fusing the two intron sequences with *GUS* in both forward and reverse directions, creating the constructs *fMdAG1I::GUS*, *rMdAG1I::GUS*, *fMdAG2I::GUS* and *rMdAG2I::GUS* (Figure 4).

**Figure 5:**
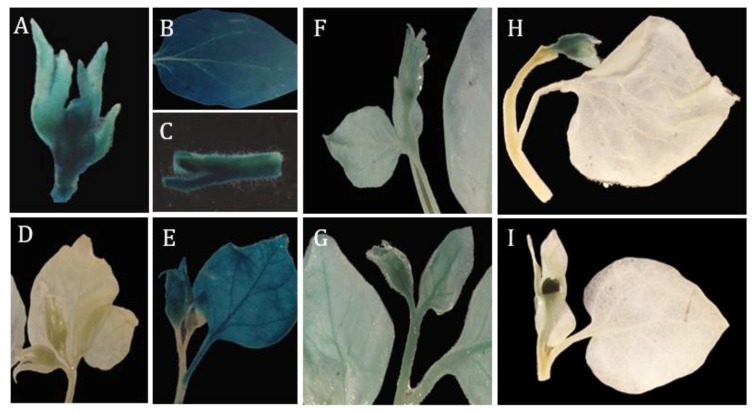
GUS staining in tobacco transformed with the four artificial promoters and *35S::GUS* control. (**A**–**C**) GUS expression using *35S::GUS* as a positive control. All organs are stained. (**D**) GUS expression in non-transformed flowers. None of the plant was stained. (**E**–**G**) GUS expression of tobacco tissues transformed with *fMdAG1I::GUS* (**E**)*, rMdAG1I::GUS* (**F**), and *fMdAG2I::GUS* (**G**), respectively. Flowers, leaves and stems all show different degrees of blue color. (**H**–**I**) Two lines of *rMdAG2I::GUS* transgenic tobacco. Only flowers are stained.

We first tested these constructs in tobacco. An *Agrobacterium*-mediated stable transformation of tobacco with the four constructs produced 28, 23, 31 and 20 lines for *fMdAG1I::GUS*, *rMdAG1I::GUS*, *fMdAG2I::GUS*, and *rMdAG2I::GUS*, respectively. We also produced eight *35S::GUS* transgenic tobacco lines. Next, we assayed the leaf, stem and flower tissues of the transgenic tobacco or non-transgenic control by histochemical staining for GUS activity. All the tested tissues exhibited GUS activity in the eight *35S::GUS* lines (Figure 5A–C and Table 1), whereas none of these tissues had GUS activity in the non-transformed control (Figure 5D).

For the *MdAG1* intron sequence, both the forward and reverse promoters exhibited *GUS* expression patterns similar to those of the *35S::GUS* plants, with GUS activity in all tested tissues. However, *fMdAG1I::GUS* caused stronger GUS activity than *rMdAG1I::GUS*, indicating that the direction of the intron sequence did affect the activity of the artificial promoter (Figure 5E,F). Of the two promoters derived from the *MdAG2* intron sequence, the forward promoter had GUS activity in both vegetative and floral organs (Figure 5G), whereas the reverse promoter had GUS activity only in floral organs, and not in vegetative organs (Figure 5H,I), suggesting a strong likelihood that *rMdAG2I::GUS* governs flower-specific expression of target genes in *Malus*. The difference in expression patterns between the forward and reverse directions of the two intron-sequences demonstrated that their activities are largely dependent on their orientation, which is consistent with our previous findings for the poplar *AG* gene [21]. The direction difference presumably leads to differences in the distances between the *cis*-regulatory sequences and transcription start sites, thus resulting in different expression patterns. In support of this, *MdAG2I* has one more LFY-binding site than *MdAG1I* at the 5′ end (Figure 3). Also, we identified only AAAC motif and TGA elements in *MdAG1I,* suggesting that *MdAG1I* may be more easily affected by environment stimuli.

Interestingly, *rMdAG2I::GUS* lines exhibited two different patterns of *GUS* expression in flower organs (Table 1). Eleven of the 20 lines (Type I) showed GUS activity in all floral organs (sepals, petals, stamens and carpels), while the remaining nine lines showed GUS activity only in stamens and carpels (Type II). Those two patterns were probably due to variable copy numbers or different transgene insertion sites among different lines. Both expression patterns would be useful in practice for breeding purposes. For instance, to produce flowerless plants without any floral organs by fusing a promoter with a toxin gene, we would need a flower-specific promoter with activity in all floral organs as Type I [21]. However, if we planned simply to produce sterile plants with normal petal development for ornamental purposes, the Type II expression pattern would be needed [33,34,35].

### 2.4. Optimization of Agrobacterium-Mediated Transient Transformation Conditions for Malus Flowers

We further tested the four constructs in two *Malus* species. Because there is no well-established stable transformation protocol for most *Malus* species, we employed *Agrobacterium*-based transient transformation to test all four constructs. The transient transformation method has the advantages of being simpler and faster than the stable transformation [36,37,38,39] and is regularly used in both *Arabidopsis* and tobacco to characterize the subcellular localization of functional genes [40,41,42]. Additionally, it has been reported that combining the clustered regularly interspersed short palindromic repeat (CRISPR)/Cas9 system and *Agrobacterium*-mediated transient transformation makes it possible to produce a target mutant with no integration of transgenes, which can be very important to accelerate the application of CRISPR technology in asexually-propagated woody plants [43,44,45,46]. In *Malus* species, especially *M. domestica*, *Agrobacterium*-mediated transient transformation has been widely used in studying fruit traits because it allows quick testing of the functions of genes associated with fruit traits, avoiding the lengthy periods associated with stable transformation methods because of the long juvenile phase of these species [47]. However, although well established for *Malus* fruit, *Agrobacterium*-mediated transient transformation is not equally successful in different sample types in *Malus* species. Therefore, we first optimized the *Agrobacterium*-mediated transient transformation system for flower tissues.

First, because the efficiency of T-DNA delivery into the host cell is one of the most important factors affecting *Agrobacterium*-mediated transient transformation, we included a vacuum infiltration step in our transient transformation system. Vacuum infiltration is reported to enhance *Agrobacterium* infection in some species, such as *Arabidopsis*, cotton and wheat [48], by improving the penetration efficiency of *Agrobacterium* cells into target plant tissues. We optimized the vacuum infiltration procedure using a *35S::GUS* construct in one representative ornamental *Malus* species, *Malus micromalus* (midget crabapple). Different plant tissues may have different tolerances of vacuum pressure due to the major variations in their cell walls and intercellular spaces. Moreover, short vacuum times may not achieve ideal results, while long vacuum times may damage the tissues [49]. Therefore, to achieve an appropriate vacuum time for flower tissues, we tested a gradient of vacuum times: 2.0 min, 3.0 min and 4.0 min, corresponding to vacuum levels of -70 kPa, -80 kPa and -85 kPa, respectively. We also observed that the length of the vacuum application affected the transformation efficiency. Therefore, we tested the results of maintaining the samples at each pressure for four different time periods: 1.0 min, 1.5 min, 2.0 min and 3.0 min. The flowers sustained more serious damage at increased vacuum levels (Table 2); in particular, the -85 kPa vacuum level was unacceptable because it caused relatively serious damage. Maintaining samples under relatively low vacuum pressure (-70 kPa) for a long time improved the staining efficiency. At -80 kPa, a large increase of the treatment time also caused tissue damages. Ultimately, we decided that a -80 kPa vacuum maintained for 1.5 min was ideal, and we used this for later experiments. By employing a modified setting, we were also able to detect GUS activity in stem materials with the *35S::GUS* construct, suggesting that, besides the floral organs, the optimized protocol is also available for stem tissues.

### 2.5. rMdAG2I::GUS Confers Flower-Specific Expression in Malus

**Figure 6:**
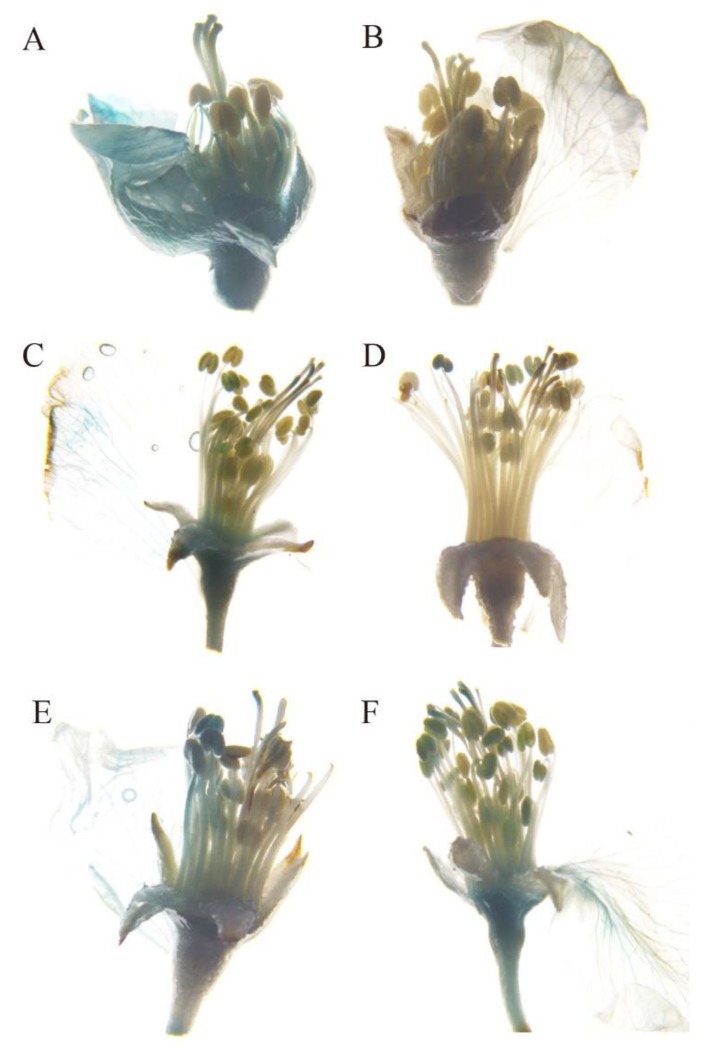
GUS staining of *Malus micromalus* with *Agrobacterium*-mediated transient transformation. (**A**) *35S::GUS*-transformed flowers (positive control). Stem and flower show blue staining. (**B**) Non-transformed flowers (negative control) have no blue staining. (**C**–**F**) *fMdAG1I::GUS*- (**C**) *rMdAG1I::GUS-* (**D**) *fMdAG2I::GUS*- (**E**) and *rMdAG2I::GUS*- (**F**) transformed flowers show blue staining.

Next, we used the optimized transformation conditions for further experiments with the four promoter constructs. We were able to detect GUS activity in all tested organs for *35S::GUS*-transformed flowers (Figure 6A), while, none GUS activity was detected for the non-transformed flowers (Figure 6B). In the *fMdAG1I::GUS* flowers, we detected GUS activity in both vegetative (stem) and floral organs (sepal, petal, stamen, pistil), which was consistent with the results in tobacco (Figure 6C, Table 3). In *rMdAG1I::GUS* flowers, we detected GUS activity in stem and stamen, but not in sepal, petal, or pistil (Figure 6D, Table 3). Notably, in tobacco, *fMdAG1I::GUS* produced higher activity than *rMdAG1I::GUS* in all instances; therefore, the undetectable activity of *rMdAG1I::GUS* in some flower organs in *M. micromalus* may be due to its global low activity. In *fMdAG2I::GUS* flowers, we detected activity in stems and flower organs (Figure 6E, Table 3), which is similar to the expression pattern of *fMdAG1I::GUS*. Because those three vectors had GUS activity in stems, they are not suitable for use as flower-specific promoters in *Malus*. Interestingly, we also detected GUS activity in the receptacles of *fMdAG1I::GUS*, *fMdAG2I::GUS* and *rMdAG2I::GUS* flowers (Table 3). The reason that the *rMdAG1I::GUS* flowers exhibited no GUS activity in receptacles may be its global low activity. Based on the gene expression data in Figure 2, the expression pattern of both *AG* genes in fruits may undergo dynamic changes. Our results suggest that the second introns of *Malus AG* genes may play some role in regulating *AG* expression during fruit development. For *rMdAG2I::GUS*, we did not detect GUS activity in stems, but we did detect GUS activity in all tested floral organs (sepal, petal, stamen and pistil) (Figure 6F, Table 3). Therefore, *rMdAG2I::GUS* has strong potential as a flower-specific promoter in *Malus*.

**Figure 7:**
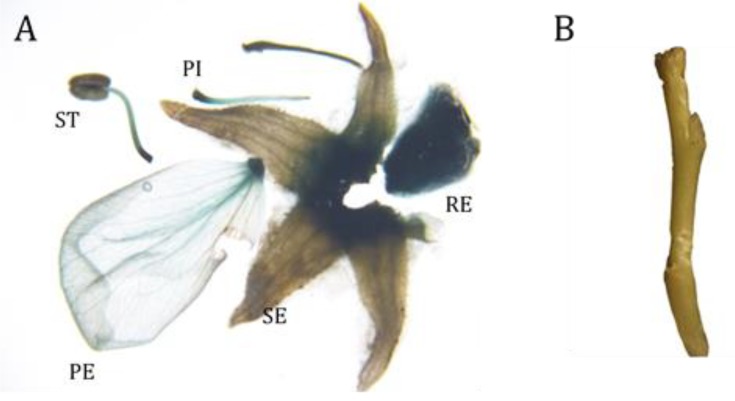
GUS staining of ‘Golden Delicious’ transformed with *rMdAG2I::GUS* through particle bombardment. (**A**) Flower organs with blue staining. RE, receptacle; SE, sepal; PE, petal; ST, stamen; PI, pistil. (**B**) Stem without blue staining.

We also tested our optimized transformation protocol with one *M. domestica* cultivar, ‘Golden Delicious’; unfortunately, the results were poor, suggesting that *Agrobacterium*-mediated transient transformation is species or material dependent. Thus, we may need to modify this transformation protocol for each new species or cultivar. Given this result, we next turned our attention to an alternative transient transformation method, particle bombardment. Although costly, this method has some important advantages, such as the absence of limitations on species or sample types [50,51,52]. Therefore, we used particle bombardment to test the flower specificity of *rMdAG2I::GUS* in ‘Golden Delicious’, using *35S::GUS* as control. With *35S::GUS*, we detected GUS activity in both vegetative organs (leaf and stem) and floral organs (sepal, petal, stamen, pistil and receptacle) (Table 4). Meanwhile, with *rMdAG2I::GUS*, we did not detect any GUS activity in vegetative organs, but we did detect GUS activity in all floral organs tested (Figure 7 and Table 4). These results further confirm the floral specificity of *rMdAG2I::GUS* in *Malus* specie.

In summary, we have successfully identified two *AG* paralogs (*MdAG1* and *MdAG2*) from *Malus*. By fusing the second-intron sequences of these two genes to the minimal *35S* promoter in both forward and reverse directions, we build four promoter constructs. Next, we demonstrated the flower specificity of the *rMdAG2I* promoter through the stable transformation of tobacco plants. We further verified its flower specificity in the *Malus* species by Agrobacterium-mediated transient transformation and particle bombardment. This newly designed *rMdAG2I* promoter thus has great potential as a means for the genetic modification of flower traits in the *Malus* species.

## 3. Materials and Methods

### 3.1. Phylogenetic Analysis

Phylogenetic analysis was conducted using MEGA 6.0 software [53]. The tree was compiled using the neighbor-joining method. Statistical reliabilities of the internal branches were assessed using 1000 bootstrap replicates. Values at nodes indicate bootstrap percentages [54].

### 3.2. RNA Extraction and qRT-PCR

The vegetative organs (roots, stems and leaves) and reproductive organs (receptacles, sepals, petals, stamens, pistils and fruits) of ‘Golden Delicious’ apples were prepared for RNA extraction. Total RNA of each sample was extracted using the cetyl trimethyl-ammonium bromide (CTAB) method. RNA quality and concentration were determined using a NANODROP 2000 spectrophotometer (Thermo Scientific, Waltham, MA, USA). The cDNA was synthesized using MLV reverse transcriptase (TakaRa Bio Inc., Dalian, China). We performed quantitative real-time PCR (qRT-PCR) analysis using the SYBR premix Ex Taq Kit (TakaRa Bio Inc., Dalian, China) in a 7500 real-time PCR system (Applied Biosystems Foster City, CA, USA).

The primer pair AG1-F (5′-GAGAGGTACAAGAAGGCAAGTGC-3′) with AG1-R (5′-CTCTTCAGGTCCTTGACAGACATAC-3′) was used for the analysis of *MdAG1* transcripts, and the pair AG2-F (5′-ATGCCTATCAAGGACCTGAAGAG-3′) with AG2-R (5′-TAGTTCCGAGAGTCGTATGGCT-3′) was used for analysis of *MdAG2* transcripts. The primer pair Actin-F (5′-TGACCGAATGAGCAAGGAAATTACT-3′) with Actin-R (5′-TACTCAGCTTTGGCAATCCACATC-3′) was used to amplify the internal reference gene, *MdActin* [47]. Data were analyzed using the 2^−ΔΔCT^ method [55]. The gene expression in each sample was calculated using the expression level of *MdActin* in the same sample. Each qRT-PCR reaction was performed with three biological replicates, and each sample was analyzed in triplicate (technical replicates).

### 3.3. Cloning of MdAG1 and MdAG2 Second-Intron Sequences 

Genomic DNA was extracted from the leaves of ‘Golden Delicious’ using a modified CTAB method [56]. Approximately 200 ng genomic DNA was used as templates for PCR amplification of the second-intron fragments from two *MdAG* genes. PCR amplification of the *MdAG1* second intron was performed using the primer pair AG1I-F (5′-CGCCTCTATGAGTATGCCAAC-3′) and AG1I-R (5′-CTTCTTGTACCTCTCAATTGTTCCT-3′) under the following conditions: 5 min at 95 °C, followed by 35 cycles of 30 s at 94 °C, 30 s at 58 °C, and 2 min at 72 °C, with a final extension of 7 min at 72 °C. PCR amplification of the *MdAG2* second intron was performed with the primer pair AG2I-F (5′-GAGTATGCCAACAACAGGTAAT-3′) and AG2I-R (5′-TGCTTTAACACTGCACATATGG-3′) under the following conditions: 5 min at 95 °C, followed by 35 cycles of 30 s at 94 °C, 30 s at 58 °C, and 2 min at 72 °C, with a final extension of 7 min at 72 °C. The amplified fragments were inserted into the pEASY-Blunt Cloning Kit, and 10–12 clones were sequenced for verification of their sequence identity.

### 3.4. Plasmid Construction 

Four chimeric promoters were created by fusing the intron sequence of either *MdAG1* or *MdAG2* to a 60-bp minimal *35S* promoter in either forward or reverse direction. Those four artificial promoters were inserted upstream of the *GUS* coding sequence to create four plasmids: *fMdAG1::GUS*, *rMdAG1::GUS*, *fMdAG2::GUS,* and *rMdAG2::GUS*. A control vector was also created containing the *GUS* coding sequence driven by a constitutively active 35S promoter (*35S::GUS*).

### 3.5. Stable Transformation of Tobacco 

Each of the four plasmids was introduced into *Agrobacterium tumefaciens* EHA105, which was subsequently used for transformation of tobacco (*Nicotiana benthamiana*) [57]. Leaf discs of tobacco (0.5 cm × 0.5 cm) were incubated for 20 min with *A. tumefaciens* (OD_600_ = 0.6) and then transferred to the Murashige and Skoog (MS) medium without hormone and antibiotics for two days of co-cultivation. After that, leaf explants were moved to a regeneration medium containing 3 mg/L 6-benzylaminopurine (6-BA), 0.2 mg/L 1-naphthaleneacetic acid (NAA), 300 mg/L cefotaxime, and 50 mg/L kanamycin. Putative transgenic shoots were moved to rooting medium containing 300 mg/L cefotaxime and 20 mg/L kanamycin. The rooting shoots were transferred to soil and grown in a greenhouse.

### 3.6. Vacuum Infiltration of Malus Micromalus Flowers

*Agrobacterium tumefaciens* EHA105 harboring each of four created constructs was prepared. Flowers of *M. micromalus* were immersed in the *Agrobacterium* suspension (OD_600_ = 1.0) and placed in a vacuum chamber. A total of 12 treatments with three vacuum treatment times (2 min, 3 min, 4 min) and four soaking times (1 min, 1.5 min, 2 min, 3 min) were used for orthogonal tests. After treatments, the samples were cultured on MS medium for two days and then subjected to GUS staining.

### 3.7. Particle Bombardment of ‘Golden Delicious’ Apple Flowers

To prepare the gold suspension, a mixture of gold powder and ethanol (60 mg:1 mL) was shaken for 1 min and centrifuged at 10,000 rpm for 10 s. The pellets were then resuspended in sterilized water. 50 µL of gold suspension, 20 μL 0.1 M spermidine, 50 μL 2.5 M CaCl_2_, and 2.5 μg DNA were used for preparing the gold/DNA mixture. After having been shaken for 3 min, the mixture was centrifuged at 10,000 rpm for 20 s. The pellets were rinsed by ethanol for two times and then re-suspended by 60 µL ethanol, and 10 μL was used for each particle bombardment. The bombardment was repeated six times for each sample. The treated samples were cultured on MS medium for two days before GUS staining.

### 3.8. GUS Staining Assays

GUS assays were performed according to a previously described protocol with modifications [21]. Samples were incubated in X-Gluc solution (100mM potassium phosphate buffer, 10 mM Na_2_EDTA, 0.5 mM K_4_Fe (CN)_6_, 0.1% Triton X-100, and 1 g/L X-Gluc, pH 7.0) at 37 °C. After 24 h of incubation, the samples were decolorized with alcohol three to five times before being photographed.

## Figures and Tables

**Table 1:** Characterization of floral specificity of *fMdAG1I::GUS*, *rMdAG1I::GUS*, *fMdAG2I::GUS*, and *rMdAG2I::GUS* transgenic tobacco.

Vectors	No. of Transgenic Lines		Plant Tissues						Floral Specificity
			Stem	Leaf	Sepal	Petal	Stamen	Carpel	
*35S::GUS*	8		+	+	+	+	+	+	No
*fMdAG1I::GUS*	28		+	+	+	+	+	+	No
*rMdAG11::GUS*	23		+	+	+	+	+	+	
*fMdAG2I::GUS*	31		+	+	+	+	+	+	
*rMdAG2I::GUS*	Type I	11	−	−	+	+	+	+	Yes
	Type II	9	−	−	−	−	+	+	

+, detectable GUS activity; −, no detectable GUS activity.

**Table 2:** Optimization of conditions for *Agrobacterium*-mediated transient transformation of *Malus* flowers using *35S::GUS* construct.

**Vacuum Time (min)**	**Soaking Time (min)**	**Condition of Treated Flowers**
2.0	1.0	No damage, No staining
2.0	1.5	No damage, Blue staining
2.0	2.0	No damage, Blue staining
2.0	3.0	No damage, Blue staining
3.0	1.0	No damage, No staining
3.0	1.5	No damage, Blue staining
3.0	2.0	Slightly damaged
3.0	3.0	Damaged
4.0	1.0	Damaged
4.0	1.5	Damaged
4.0	2.0	Damaged
4.0	3.0	Damaged

**Table 3:** Characterization of floral specificity of *fMdAG1I::GUS*, *rMdAG1I::GUS*, *fMdAG2I::GUS* and *rMdAG2I::GUS* in *Malus micromalus* with *Agrobacterium*-mediated transient transformation.

	Organs	Stem	Sepal	Petal	Stamen	Pistil	Receptacle
Vectors							
*35S::GUS*		+	+	+	+	+	+
*fMdAG1I::GUS*		+	+	+	+	+	+
*rMdAG1I::GUS*		+	−	−	+	−	−
*fMdAG2I::GUS*		+	+	+	+	+	+
*rMdAG2I::GUS*		−	+	+	+	+	+

+, detectable GUS activity; −, no detectable GUS activity.

**Table 4:** Characterization of the activity of *rMdAG2I* in ‘Golden Delicious’ through particle bombardment.

Organ	Stem	Leaf	Sepal	Petal	Stamen	Pistil	Receptacle
*35S::GUS*	+	+	+	+	+	+	+
*rMdAG2I::GUS*	−	−	+	+	+	+	+

+, detectable GUS activity; −, no detectable GUS activity.

## References

[1] Phipps J.B., Robertso K.R., Smith P.G., Rohrer J.R. (1988). A checklist of the subfamily Maloideae (Rosaceae). Can. J. Bot..

[2] Lisandru T.T., Adrian FÜstÖs A., Adelina DumitraŞ A., Viorel Mitre V. (2017). Flower development of ornamental Crabapple according to BBCH scale. Bulletin UASVM Hort..

[3] Del Duca S., Aloisi I., Parrotta L., Cai G. (2019). Cytoskeleton, Transglutaminase and gametophytic self-incompatibility in the Malinae (Rosaceae). Int. J. Mol. Sci..

[4] Broothaerts W., Janssens G.A., Proost P., Broekaert W.F. (1995). cDNA cloning and molecular analysis of two self-incompatibility alleles from apple. Plant Mol. Biol..

[5] Mir J., Ahmed N., Singh D.B., Sheemar G., Hamid A., Zaffer S., Shafi W. (2016). Molecular identification of S-alleles associated with self-incompatibility in apple (Malus spp.) genotypes. Indian J. Agric. Sci..

[6] Garratt M.P., Breeze T.D., Boreux V., Fountain M.T., McKerchar M., Webber S.M., Coston D.J., Jenner N., Dean R., Westbury D.B. (2016). Apple pollination: Demand depends on variety and supply depends on pollinator identity. PLoS ONE.

[7] Sapir G., Baras Z., Azmon G., Goldway M., Shafir S., Allouche A., Stern E., Stern R.A. (2017). Synergistic effects between bumblebees and honey bees in apple orchards increase cross pollination, seed number and fruit size. Sci. Hortic..

[8] Russo L., Park M.G., Blitzer E.J., Danforth B.N. (2017). Flower handling behavior and abundance determine the relative contribution of pollinators to seed set in apple orchards. Agric. Ecosyst. Environ..

[9] Robbie F.A., Atkinson C.J. (1994). Wood and tree age as factors influencing the ability of apple flowers to set fruit. J. Hortoc. Sci..

[10] Kelsey D.F., Brown S.K. (1992). ‘McIntosh Wijcik’: A columnar mutation of ‘McIntosh’ apple proving useful in physiology and breeding research. J. Am. Pomol. Soc..

[11] Fisher D.V. (1995). The ‘Wijcik Spur McIntosh’. Fruit Varieties J..

[12] Kim D.H., Park S., Lee J.Y., Ha S.H., Lim S.H. (2019). Enhancing flower color through simultaneous expression of the *B-peru* and *mPAP1* transcription factors under control of a flower-specific promoter. Int. J. Mol. Sci..

[13] Verdonk J.C., Shibuya K., Loucas H.M., Colquhoun T.A., Underwood B.A., Clark D.G. (2008). Flower-specific expression of the *Agrobacterium tumefaciens* isopentenyltransferase gene results in radial expansion of floral organs in Petunia hybrida. Plant Biotechnol. J..

[14] Liao X., Guo X., Wang Q., Wang Y., Zhao D., Yao L., Wang S., Liu G., Li T. (2017). Overexpression of MsDREB6.2 results in cytokinin-deficient developmental phenotypes and enhances drought tolerance in transgenic apple plants. Plant J..

[15] Li Y., Hagen G., Guilfoyle T.J. (1992). Altered morphology in transgenic tobacco plants that overproduce cytokinins in specific tissues and organs. Dev. Biol..

[16] Werner T., Holst K., Pors Y., Guivarc’h A., Mustroph A., Chriqui D., Grimm B., Schmülling T. (2008). Cytokinin deficiency causes distinct changes of sink and source parameters in tobacco shoots and roots. J. Exp. Bot..

[17] Yanofsky M.F., Ma H., Bowman J.L., Drews G.N., Feldmann K.A., Meyerowitz E.M. (1990). The protein encoded by the *Arabidopsis* homeotic gene agamous resembles transcription factors. Nature.

[18] Drews G.N., Bowman J.L., Meyerowitz E.M. (1991). Negative regulation of the *Arabidopsis* homeotic gene *AGAMOUS* by the *APETALA2* product. Cell.

[19] Liu Z., Liu Z. (2008). The second intron of *AGAMOUS* drives carpel- and stamen-specific expression sufficient to induce complete sterility in *Arabidopsis*. Plant Cell Rep..

[20] Yang Y., Singer S.D., Liu Z. (2010). Two similar but distinct second intron fragments from tobacco *AGAMOUS* homologous confer identical floral organ-specific expression sufficient for generating complete sterility in plants. Planta.

[21] Li W., Hu W., Feng C., Chen L., Zhuang W., Katin-Grazzini L., McAvoy R.J., Guillard K., Li Y. (2016). An *AGAMOUS* intron-driven cytotoxin leads to flowerless tobacco and produces no detrimental effects on vegetative growth of either tobacco or poplar. Plant Biotechnol. J..

[22] Van der Linden C.G., Vosman B., Smulders M.J.M. (2002). Cloning and characterization of four apple *MADS* box genes isolated from vegetative tissue. J. Exp. Bot..

[23] Klocko A.L., Borejsza-Wysocka E., Brunner A.M., Shevchenko O., Aldwinckle H., Strauss S.H. (2016). Transgenic suppression of *AGAMOUS* genes in apple reduces fertility and increases floral attractiveness. PLoS ONE.

[24] Wang H.Z., Hu B., Chen G.P., Shi N.N., Zhao Y., Yin Q.C., Liu J.J. (2008). Application of *Arabidopsis AGAMOUS* second intron for the engineered ablation of flower development in transgenic tobacco. Plant Cell Rep..

[25] Bowman J.L., Drews G.N., Meyerowitz E.M. (1991). Expression of the *Arabidopsis* floral homeotic gene *AGAMOUS* is restricted to specific cell types late in flower development. Plant Cell.

[26] Ito T., Ng K.H., Lim T.S., Yu H., Meyerowitz E.M. (2007). The Homeotic Protein AGAMOUS Controls Late Stamen Development by Regulating a Jasmonate Biosynthetic Gene in *Arabidopsis*. Plant Cell.

[27] Eccher G., Ferrero S., Populin F., Colombo L., Botton A. (2014). Apple (*Malus domestica* L. Borkh) as an emerging model for fruit development. Plant Biosyst..

[28] Klee H.J., Giovannoni J.J. (2011). Genetics and Control of Tomato Fruit Ripening and Quality Attributes. Annu. Rev. Genet..

[29] Lescot M., Déhais P., Thijs G., Marchal K., Moreau Y., Van D.P.Y., Rouzé P., Rombauts S. (2002). PlantCARE, a database of plant *cis*-acting regulatory elements and a portal to tools for in silico analysis of promoter sequences. Nucleic. Acids. Res..

[30] Busch M.A., Bomblies K., Weigel D. (1999). Activation of a floral homeotic gene in *Arabidopsis*. Science.

[31] Hong R., Hamaguchi L., Busch M.A., Weigel D. (2003). Regulatory Elements of the Floral Homeotic Gene *AGAMOUS* Identified by Phylogenetic Footprinting and Shadowing. Plant Cell..

[32] Maizel A., Busch M.A., Tanahashi T., Perkovic J., Kato M., Mitsuyasu Hasebe M., Weigel D. (2005). The floral regulator *LEAFY* evolves by substitutions in the DNA binding domain. Science.

[33] Liu M., Li W., Zhao G., Fan X., Long H., Fan Y., Shi M., Tan X., Zhang L. (2019). New insights of salicylic acid into stamen abortion of female flowers in tung tree (*Vernicia fordii*). Front. Genet..

[34] Yue Y., Yang D., Sun J., Peng H., Yin C., Guo R., Ning G., Hu H. (2016). A novel *PhLRR* gene promoter is sufficient for engineering male sterility in petunia. Plant Mol. Biol. Rep..

[35] Tang L., Chu H., Yip W.K., Yeung E.C., Lo C. (2009). An anther-specific dihydroflavonol 4-reductase-like gene (*DRL1*) is essential for male fertility in *Arabidopsis*. New Phytol..

[36] Shang Y., Schwinn K.E., Bennett M.J., Hunter D.A., Waugh T.L., Pathirana N.N., Brummell D.A., Jameson P.E., Davies E.M. (2007). Methods for transient assay of gene function in floral tissues. Plant Methods.

[37] Wen X., Wang J., Zhang D., Wang Y. (2019). A gene regulatory network controlled by *BpERF2* and *BpMYB102* in Birch under drought conditions. Int. J. Mol. Sci..

[38] Chevreau E., Dousset N., Joffrion C., Richer A., Charrier A., Vergne E. (2019). Agroinfiltration is a key factor to improve the efficiency of apple and pear transformation. Sci. Hortic..

[39] Lin Q., Li Z., Zhang L., Tan X., Long H., Wu L. (2016). High-efficiency regeneration of seedlings from hypocotyl explants of tung tree (*Vernicia fordii*). Int. J. Agric. Biol..

[40] Tsuda K., Qi Y., Nguyen V.L., Bethke G., Tsuda Y., Glazebrook J., Katagiri F. (2012). An efficient *Agrobacterium*-mediated transient transformation of Arabidopsis. Plant J..

[41] Hwang H., Yu M., Lai E. (2017). *Agrobacterium*-mediated plant transformation: Biology and applications. Arabidopsis Book.

[42] Liu K., Yang Q., Yang T., Wu Y., Wang G., Yang F., Wang R., Lin X., Li G. (2019). Development of *Agrobacterium*-mediated transient expression system in Caragana intermedia and characterization of *CiDREB1C* in stress response. BMC Plant Biol..

[43] Xu R.F., Li H., Qin R.Y., Wang L., Li L., Wei P.C., Yang J.B. (2014). Gene targeting using the *Agrobacterium tumefaciens*-mediated CRISPR-Cas system in rice. Rice.

[44] Jia H.G., Wang N. (2014). Targeted genome editing of sweet orange using Cas9/sgRNA. PLoS ONE.

[45] Jia H., Zhang Y., Orbović V., Xu J., White F.F., Jones J.B., Wang N. (2017). Genome editing of the disease susceptibility gene *CsLOB1* in citrus confers resistance to citrus canker. Plant Biotechnol. J..

[46] Nishitani C., Hirai N., Komori S., Wada M., Okada K., Osakabe K., Yamamoto T., Osakabe Y. (2016). Efficient genome editing in apple using a CRISPR/Cas9 system. Sci. Rep-UK.

[47] Han Z.Y., Hu Y.N., Lv Y.D., Rose J.K.C., Sun Y.Q., Shen F., Wang Y., Zhang X.Z., Xu X.F., Wu T. (2018). Natural variation underlies differences in *ETHYLENE RESPONSE FACTOR17* activity in fruit peel degreening. Plant Physiol..

[48] Yan H.X., Fu D.Q., Zhu B.Z., Liu H.P., Shen X.Y., Luo Y.B. (2012). Sprout vacuum-infiltration: A simple and efficient agroinoculation method for virus-induced gene silencing in diverse solanaceous species. Plant Cell Rep..

[49] Sapers G.M., Garzarella L., Pilizota V. (1990). Application of browning inhibitors to cut apple and potato by vacuum and pressure infiltration. Food Sci..

[50] Dai S., Zheng P., Marmey P., Zhang S., Tian W., Chen S., Beachy R.N. (2001). Comparative analysis of transgenic rice plants obtained by *Agrobacterium*-mediated transformation and particle bombardment. Mol. Breeding.

[51] Azuma M., Morimoto R., Hirose M., Morita Y., Hoshino A., Iida S., Oshima Y., Mitsuda N., Takagi M.O., Shiratake K. (2016). A petal-specific *InMYB1* promoter from Japanese morning glory: A useful tool for molecular breeding of floricultural crops. Plant Biotechnol. J..

[52] Zapata D.G., Vargas J.M.S., Osorio K.S.M., Manjarrez E.S., Flores J.L.C., Salvador M.D.C.O., Figueroa N.V.D., Nogué F., Corona A.B. (2019). Efficient eiting of the nuclear *APT* reporter gene in *Chlamydomonas reinhardtii* via expression of a CRISPR-Cas9 module. Int. J. Mol. Sci..

[53] Tamura K., Stecher G., Peterson D., Filipski A., Kumar S. (2013). MEGA6: Molecular Evolutionary Genetics Analysis Version 6.0. Mol. Biol. Evol..

[54] Saitou N., Nei M. (1987). The neighbor-joining method: A new method for reconstructing phylogenetic trees. Mol. Biol. Evol..

[55] Livak K.J., Schmittgen T.D. (2011). Analysis of relative gene expression data using real-time quantitative PCR and the 2^−ΔΔCT^ Method. Methods.

[56] Porebski S., Bailey L.G., Baum B.R. (1997). Modification of a CTAB DNA extraction protocol for plants containing high polysaccharide and polyphenol components. Plant Mol. Biol. Rep..

[57] Xu X., Li X., Hu X., Wu T., Wang Y., Xu X., Zhang X., Han Z. (2017). High miR156 Expression Is Required for Auxin-Induced Adventitious Root Formation via *MxSPL26* Independent of *PINs* and *ARFs* in *Malus xiaojinensis*. Front. Plant Sci..

